# The Clinicopathological Features of Mediastinal Tuberculous Lymphadenitis in Cancer Patients and the Diagnostic Role of Endobronchial Ultrasound

**DOI:** 10.7759/cureus.15837

**Published:** 2021-06-22

**Authors:** Usman Khalid, Muhammad J Akram, Faheem M Butt, Mohammad B Ashraf, Faheem Khan

**Affiliations:** 1 Internal Medicine, Shaukat Khanum Memorial Cancer Hospital and Research Centre, Lahore, PAK

**Keywords:** tb, cancer, ebus, tbna, mediastinal lymph nodes

## Abstract

Introduction

Mediastinal lymphadenopathy in cancer patients can be of both malignant and non-malignant (including infectious) etiology. Tuberculosis (TB) is an important differential in this regard, particularly in regions with high TB endemicity.

Objectives

To determine the incidence and clinical characteristics of mediastinal tuberculous lymphadenitis (MTBLA) in cancer patients of a TB-endemic region, and the diagnostic role of endobronchial ultrasound (EBUS) guided transbronchial needle aspiration (TBNA) in such patients, utilizing both cytopathological and microbiological parameters for diagnosing TB.

Materials and methods

We retrospectively analyzed the relevant clinical data of all cancer patients diagnosed with MTBLA after undergoing EBUS-TBNA at our center, between July 2013 till July 2018 (total five years). The diagnostic yield, sensitivity and specificity of cytopathological and microbiological investigations (including TB culture and *Mycobacterium tuberculosis *Gene Xpert assay) for diagnosis of MTBLA were determined.

Results

Of the total 493 cancer patients, MTBLA was diagnosed in 54 (11%), with mean age of 48 ± 12 years, and predominantly male gender (59.3%). Thirty-three (61.1%) patients were clinically asymptomatic at the time of presentation, while cough was reported by 13 (24.7%) patients and weight loss, shortness of breath and fever by only six (11.1%), six (11.1%) and five (9.2%) patients, respectively. Total 53% had an underlying gastrointestinal malignancy. Chest imaging revealed bilateral versus unilateral hilar lymph node enlargement in 32 (59.3%) against 22 (40.7%) patients, respectively, while only 14 (25.9%) had accompanying lung parenchymal findings. Granulomatous TBNA cytology was detected in 41 (77.3%) patients, giving a diagnostic yield of 70.3% for MTBLA, with an estimated sensitivity and specificity of 79.2% and 99%, respectively. TB culture and Gene Xpert had a respective sensitivity of 48% and 53%, with the combined diagnostic yield of 64.8%. Treatment response was achieved in 51 (94%) patients, based on which EBUS was estimated to have sensitivity and specificity of 89% and 99% respectively, with no reported complications.

Conclusion

Mediastinal TB can have diverse manifestations among cancer patients and can often be clinically occult, with overlapping radiological impressions. EBUS-TBNA can serve as a safe and reliable diagnostic tool in this regard.

## Introduction

In cancer patients, mediastinal lymphadenopathy can be attributed to both infectious and non-infectious (including malignant) etiologies [1]. It thus bears important implications on the staging and treatment of the patient. In regions where it is endemic, tuberculosis (TB) can possibly be singled out as the most common infectious etiology [2]. TB remains a burgeoning global health concern, with the WHO estimating 10.0 million new cases worldwide, and approximately 1.4 million deaths related to this infection in 2018, with South East Asia being the most affected demographic region [3]. It has been determined to be an independent risk factor for morbidity, increased hospitalization costs and mortality in cancer patients [4]. Tuberculosis is known to inflict cancer patients of all kinds, and has protean manifestations, as in non-cancer patients [5,6]. Untreated tuberculosis can be a cause of further clinical deterioration in cancer patients, particularly those receiving radical chemotherapy, radiotherapy and immunotherapy, with the latter particularly increasing the vulnerability to disseminated infections due to immune suppression [6,7].

Mediastinal tuberculous lymphadenitis (MTBLA) is a challenging manifestation of TB, especially in terms of diagnosis. It shows a wide spectrum of clinical and radiological presentations [8], which at times may mimic malignant disease. If misdiagnosed as cancer, this curable infection may remain untreated, and possibly progress [9]. The incidence of MTBLA is estimated to be significantly high in cancer patients belonging to regions with high TB endemicity [10]. Endobronchial ultrasound (EBUS) guided transbronchial needle aspiration (TBNA) is regarded as a minimally invasive, yet accurate diagnostic tool for MTBLA, with the estimated sensitivity and specificity ranging between 83-95% and 92-99%, respectively [11-14].

The cytopathological diagnosis of MTBLA on fine needle aspirate (FNA) is based on presence of granulomatous cytology, while positive TB culture is the microbiological gold standard. However, with evolving experience, polymerase chain reaction (PCR) and Gene Xpert assays for *Mycobacterium tuberculosis* have acquired attention and approval, more so because of the rapidity of the results, compared to TB culture [15,16]. The aforementioned studies on EBUS corroborate that nodal cytopathology has a higher diagnostic yield compared to TB culture, and is predominantly of necrotizing granulomatous type [11-16]. However, these studies have been conducted on predominantly non-cancer patients. It is unknown whether a baseline malignancy and subsequent treatment with chemo-radiation significantly alters the nodal cytopathology, making cytological diagnosis ambiguous, whereby the diagnostic value of culture and Gene Xpert would naturally increase. Furthermore, the clinical and radiological manifestations of mediastinal tuberculosis have been less often studied in a population of cancer patients, in whom overlap and concurrence is a concern, particularly in regions with high TB endemicity.

Our tertiary care cancer setup, located in a region with high prevalence of TB, is at the cusp of the diagnostic dilemma. Through this study, we aim to evaluate and present our clinical experience regarding diagnosis and treatment of mediastinal tuberculosis, in patients with active or treated cancer, with focus on both clinical and radiological presentations of MTBLA, as well as the diagnostic utility of EBUS-guided transbronchial needle aspiration (TBNA). Additionally, we wished to evaluate the individual diagnostic yield and sensitivity of cytopathology and microbiological investigations (including TB culture and Gene Xpert) on TBNA aspirates in such patients. The study is expected to provide useful insight to the occurrence and diagnostic characteristics of MTBLA in cancer patients in our populations setting. Both TB and cancer are important health concerns in Pakistan, as well as the surrounding regions, and an improved knowledge will certainly pave way for evidence-based practice and management of this condition.

## Materials and methods

The study design was retrospective cross-sectional and the due exemption was granted by the Institutional Review Board of the institution. We acquired the data of all patients (total 620) who underwent EBUS-TBNA at our tertiary care cancer center, from July 2013 until December 2018 (total five years). By conducting an initial review of the electronic medical records of each patient, we shortlisted all cancer patients in whom the clinical diagnosis of MTBLA was established subsequent to EBUS-TBNA. The relevant clinical, microbiological and radiological information was then obtained through a detailed review of the same electronic medical records of these patients.

Diagnostic imaging of the chest with contrast enhanced computed tomography (CT) scans had been performed in each patient to identify mediastinal lymphadenopathy prior to EBUS-TBNA, followed by clinical evaluation by the interventional pulmonologist. Baseline coagulation and renal profile was checked for each patient and the risk for sedation was estimated using the Mallampati score. All patients underwent EBUS-TBNA under routine intravenous sedation, using Midazolam and Fentanyl, along with topical sprays of Lignocaine 4% and Lignocaine 2% for local anesthetic effect. EBUS was performed using an Olympus scope model UC260FW with convex probe and a 22-Gauge FNA needle. The adequacy of all TBNA samples was assessed at the spot, by a rapid on-site evaluation (ROSE) team, comprising trained cytopathologists, as per the globally recommended practice [17]. TBNA samples underwent acid fast bacillus (AFB) smear staining, TB culture (using Bactec and Lowenstein Jensen media) and cell block preparation on all specimens, and* Mycobacterium tuberculosis* (MTB) Gene Xpert assay where available.

Criteria for the cytological diagnosis of tuberculosis were the presence of granulomatous inflammation, with or without caseous necrosis, presence of epithelioid cells or giant cells [18]. Positive microbiology results of *Mycobacterium tuberculosis* Gene Xpert and TB culture were treated as diagnostic for TB and considered to be true positives for each of these parameters while estimating their respective sensitivity and positive predictive values. All patients ultimately diagnosed with TB received a six-month standard anti-tuberculous treatment (ATT) regime, as per World Health Organization (WHO) recommendations [19]. Treatment response was defined as clinical improvement and/or reduction in the size of mediastinal lymphadenopathy on the repeat contrast enhanced CT chest, at the completion of treatment. The patients’ electronic medical records were followed for up to one year after the completion of ATT. The respective sensitivity, specificity, positive and negative predictive values for all cytopathological and microbiological investigations were computed. For the purpose of this study, only the patients who did not respond to the standard treatment regime, in the absence of non-compliance to treatment or identified drug resistance, were considered as "false positives." Lastly, in the remaining cancer patients who did not meet the diagnostic criteria for MTBLA on TBNA specimens, a detailed review of the medical records was similarly extended to a period of one year post EBUS, to determine the number of patients diagnosed with MTBLA afterwards through further invasive investigation (or in other words false negatives for EBUS-TBNA).

## Results

**Figure 1:**
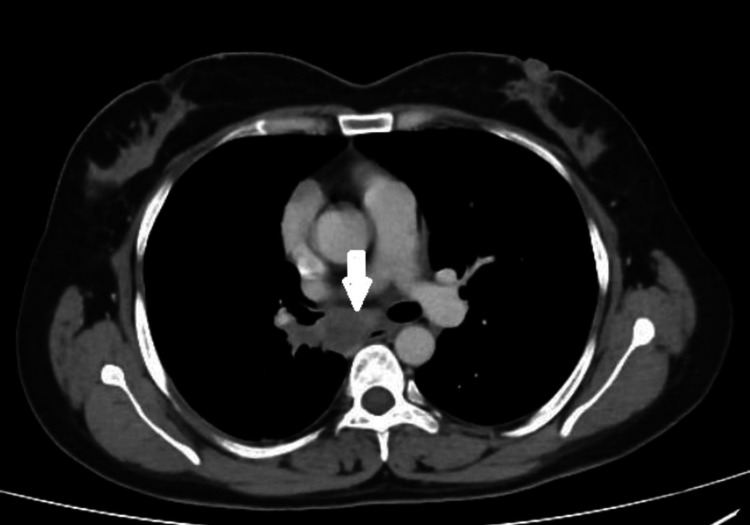
A section of the contrast enhanced CT Chest of a patient diagnosed with breast carcinoma, showing enlarged subcarinal or station 7 lymph node (white arrow)

**Figure 2:**
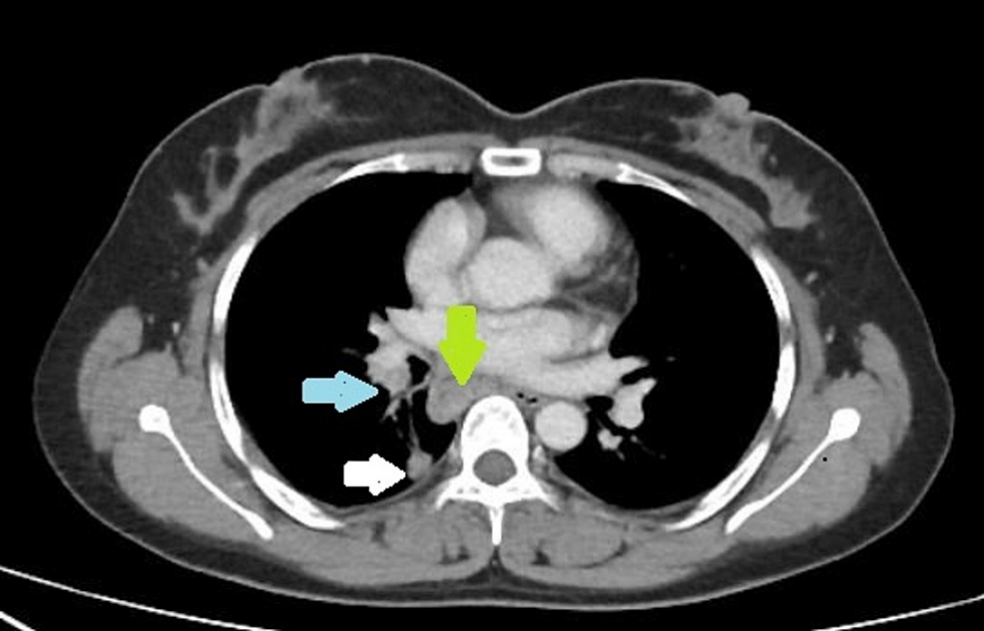
A section from the contrast enhanced CT Chest of a patient diagnosed with adenocarcinoma lung, showing right lower lung nodule (white arrow) and enlarged right hilar (blue arrow) and subcarinal (green arrow) lymph nodes.

Fifty-four (11.1%) of the total 493 cancer patients who underwent EBUS-TBNA during the stipulated period were diagnosed with MTBLA. At the time of presentation, 33 (61.1%) patients were asymptomatic. Among those showing clinical symptoms, cough was the most common symptom, reported by 13 (24.7%) patients, whereas only five (9.3%) had a documented fever and 12 (22.2%) recalled a positive TB contact. On contrast enhanced CT chest, 32 (59.2%) patients had bilateral hilar and mediastinal lymphadenopathy, while 14 (25.9%) had pulmonary infiltrates. The demographic, clinical and radiological features of the patients are presented in detail in Table 1, and sections from contrast enhanced CT chest from two separate patients, each showing enlarged mediastinal lymph nodes and relevant findings are shown in Figure 1 and Figure 2.

**Table 1:** Key demographics, clinical and radiological characteristics of patients diagnosed with mediastinal tuberculosis shown as number and percentage of total or as otherwise stated. † Mediastinal tuberculous lymphadenitis ‡ Standard deviation

Variable	Number of patients (%)
Number of patients diagnosed MTBLA^†^	54 (11.1%)
Gender	
Male	32 (59.3%)
Female	22 (40.7%)
Mean Age (years ± SD ^‡^)	48 ± 12
Clinical symptoms	
Cough	13 (24.7%)
Weight loss	6 (11.1%)
Shortness of breath	6 (11.1%)
Fever	5 (9.2%)
Anorexia	2 (3.7%)
Hemoptysis	2 (3.7%)
Chest pain	1 (1.8%)
Positive TB contact	12 (22.2%)
Asymptomatic	33 (61.1%)
Radiological features (on contrast enhanced CT Chest)	
Bilateral hilar and mediastinal lymphadenopathy	32 (59.3%)
Unilateral hilar/mediastinal lymphadenopathy	22 (40.7%)
Extra thoracic lymphadenopathy	4 (7.4%)
Lymph node calcification	5 (9.3%)
Pulmonary and pleural findings	
Lung infiltrates	14 (25.9%)
Calcified nodules	8 (14.8%)
Pleural thickening/effusion	4 (7.4%)
Lung cavitation	1 (1.9%)

Positron emission tomography (PET) scan had been performed in only 16 of the patients, with a mean standard uptake value (SUV) of the lymph nodes being 5.96 ± 3.16. Simultaneous bronchial washings had been obtained in 12 (22.2%) patients, establishing microbiological diagnosis of TB in just five (9.3%) patients through this method. The total number of lymph nodes sampled in our patient population with MTBLA was 68, with the mean size of lymph nodes being 2.06 ± 2.04 cm. Station 7 (subcarinal station) was the most common lymph node station sampled [number of times sampled (n) = 27, 39.7%] followed by station R4 or right paratracheal (n = 25, 36.7%). The complete details of the lymph nodes sampled and the resultant TBNA cytology in each is shown in Table 2.

**Table 2:** The total number of lymph node stations sampled and the respective TBNA cytology. TBNA: Transbronchial needle aspiration

Lymph Node Station	Number of times sampled	Granulomatous Cytology	Reactive Cytology	Malignant Cytology	Inadequate Cytology
Station 7	27	17 (63%)	09 (33.33%)	0	01 (13%)
Station R4	25	19 (76%)	04 (16%)	01 (4%)	01 (5.9%)
Station 11L	07	03 (42.9%)	03 (42.9%)	0	01 (14.2%)
Station L4	04	01 (25%)	02 (50%)	0	01 (25%)
Station R11	03	02 (66.7%)	01 (33%)	0	0
Station R10	02	01 (50%)	0	0	01 (50%)
Total	68	43 (63.2%)	19 (27.9%)	01 (1.5%)	05 (7.3%)

The etiology-wise data showed that gastrointestinal (GI) malignancies cumulatively accounted for the majority of the cases. Colorectal carcinomas accounted for 12% of the cases while gastric and esophageal carcinomas accounted for 7% and 8%, respectively. Cumulatively, GI malignancies accounted for 53% of the total cases. The cancer-wise number of cases are shown in Table 3.

**Table 3:** Number and percentage cases of MTBLA per individual cancer type. *MTBLA: Mediastinal tuberculous lymphadenitis

Malignancy		Total Cases (Out of 493)			Number of patients with MTBLA* (Out of 54)			Per individual cancer TB cases	
Colorectal Carcinomas		43 (8.7%)			14 (25.9%)			32.6% (14/43)	
Esophageal Carcinoma		66 (13%)			08 (14.8%)			12.1% (08/66)	
Gastric Carcinoma		35 (7%)			07 (13%)			20% (07/35)	
Breast Carcinoma		59 (11%)			04 (7.4%)			6.8% (04/59)	
Ovarian Carcinoma		11 (2%)			03 (5.6%)			27.3% (03/11)	
Endometrial Carcinoma		10 (1%)			03 (5.6%)			30% (03/10)	
Papillary Carcinoma Thyroid		09 (1%)			03 (5.6%)			33.3% (03/09)	
Cervical Carcinoma		13 (2%)			03 (5.6%)			23% (3/13)	
Hodgkin’s Lymphoma		38 (6%)			02 (3.7%)			5.2% (2/38)	
Chronic Myeloid Leukemia		02 (0.3%)			01 (1.9%)			50% (01/02)	
Adenocarcinoma Lung		26 (4%)			01 (1.9%)			4% (01/26)	
Non-Hodgkin’s Lymphoma		26 (94%)			01 (1.9%)			4% (01/26)	
Bladder Carcinoma		12 (2%)			01 (1.9%)			8% (01/12)	
Ewing’s Sarcoma		03 (0.4%)			01 (1.9%)			33% (01/03)	
Pancreatic Carcinoma		10 (1%)			01 (1.9%)			10% (01/10)	
Mucoepidermoid Tumour		02 (0.3%)			01 (1.9%)			50% (01/02)	

In 54 patients who were ultimately diagnosed with MTBLA, the TBNA cytology was granulomatous in 41 (77.3%) patients, reactive in nine (17%) and malignant in one (1.9%) patient. Notably, the cytology was reported “inadequate” for cytological diagnosis in three (3.7%) patients, revealing only necrosis. The malignant cytology was detected in a patient with breast carcinoma, with positive immunostaining for GATA-3 binding protein. The same patient had a positive TB culture. The TBNA cytology was classified as necrotizing granulomatous in 14/41 (34%) patients. Furthermore, 19 patients with granulomatous inflammation were diagnosed MTBLA on clinical grounds, despite negative microbiology results. In three such patients, the treatment response was poor, and the diagnosis was subsequently (after completion of anti-tuberculous therapy) revised to sarcoidosis. They were thus assumed to be false positives for the purpose of this study. Thus, the diagnostic yield of granulomatous cytology alone for the diagnosis of MTBLA was 70.3% (after exclusion of three false positives) in our study population, with an estimated sensitivity, specificity, positive predictive value (PPV) and negative predictive value (NPV) of 79.2%, 99%, 92.7% and 97%, respectively. Of note, the positive predictive value was 100% with necrotizing granulomatous inflammation. Compared to cytology, the diagnostic yield for positive microbiology (combining the yield of TB culture and gene Xpert) for diagnosis of MTBLA in our patients was 64.8% (total 35 patients had positive microbiological evidence of TB). The details of microbiological results with respect to the cytopathology of lymph nodes are shown in Table 4.

**Table 4:** Microbiology (Gene Xpert and TB culture) results with respect to TBNA cytology shown as number and percentage of total. TBNA: Transbronchial needle aspiration

TBNA cytology	Number of patients	Positive TB culture	Positive MTB Gene Xpert (performed in 30 patients)	Negative TB culture & Gene Xpert
Necrotizing granulomatous	14 (25.9%)	04	04	08 (57.1%)
Non-necrotizing granulomatous	27 (50%)	11	04	11 (40.7%)
Reactive	09 (16.7%)	07	05	0
Malignant	01 (1.9%)	01	0	0
Inadequate	03 (5.6%)	03	03	0
Total	54	26	16	19 (35.2%)

Overall, MTB gene Xpert was performed in total 310 (63%) out of the 493 cancer patients, and 30/54 (55.6%) patients diagnosed with MTBLA, due to availability of the test in the hospital only in the later two-third of the study period. It was positive in 16 patients (true positives for Gene Xpert), thus having estimated sensitivity 53.3%, specificity 100%, PPV 100% and NPV 95% (total 280 true negatives and 14 false negatives). On the other hand, TB culture was performed in all patients in the study. The TB culture was positive in 26 (48%) patients (true positives) and negative in 28 (false negative) MTBLA patients. The estimated sensitivity, specificity, PPV and NPV of TB culture for diagnosis of MTBLA was thus 48%, 100%, 100% and 94%, respectively. Notably, eight patients with positive MTB gene Xpert had a negative TB culture, while six patients had positive TB culture and negative MTB Gene Xpert. Drug sensitivity testing did not reveal any case of drug-resistant TB, while treatment was completed with compliance in all patients. No procedure-related complication or adverse event was reported in any patient in short- and long-term follow-up. Anti-tuberculous therapy-associated adverse effects were reported in three patients overall; transient hepatitis in one and skin rash in two patients. After the completion of the standard treatment, clinical and radiological response was seen in 51 (94%) patients. The clinical records of these patients were followed for one year subsequent to treatment completion, and revealed no evidence of recurrence of symptoms or suspicion of an alternative diagnosis (true positives for EBUS).

The medical records of the remaining 439 patients were similarly followed for a period of one year subsequent to the procedure (EBUS-TBNA). Among these patients, histopathological and microbiological diagnosis of MTBLA was established in two via surgical lymph node biopsy through video-assisted thoracoscopic surgery (VATS). Additionally, TB was diagnosed in one patient with transbronchial lung biopsy and in one patient with endoscopic ultrasound (EUS) guided fine needle aspiration (FNA) of mediastinal lymph node. Two patients with mediastinal lymphadenopathy had positive microbiological work up of TB on bronchial washings, while the same was negative for TB in these patients on TBNA aspirate. Assuming all of the above six patients as false negatives for EBUS, and together with the aforementioned three false positives, the overall sensitivity, specificity, PPV and NPV of EBUS TBNA for diagnosis of MTBLA is estimated to be around 89%, 99%, 94.4% and 98.6%.

## Discussion

Mediastinal lymphadenopathy in cancer patients often masquerades as metastatic disease; however, TB remains an important differential. This retrospective study focused on the diagnostic role of EBUS-TBNA for the diagnosis of MTBLA, specifically in cancer patients with mediastinal lymphadenopathy, belonging to a TB-endemic demographic, while also highlighting its clinical and radiological manifestations, and prevalence among such patients with respect to different cancer etiologies. The study determines that clinical symptoms in such patients are quite often minimal, while the radiographic manifestations quite often mimic malignant disease, including the nodal SUV on sophisticated PET imaging. Diagnostic testing via EBUS-TBNA appears to be safe and sufficiently accurate. The study does not determine any significant burden of drug-resistant TB in this study population.

In a study conducted on non-cancer patients by Jacob et al., 45% of patients diagnosed with MTBLA were clinically asymptomatic, and majority had unilateral (mainly right paratracheal) lymph node enlargement on CT imaging [8]. Navani et al. reported fever in 49%, cough in 60%, weight loss in 46% of their patients, while only 22% were asymptomatic [11]. In our study, the larger majority (61%) of MTBLA patients were clinically asymptomatic at the time of presentation. Overall, 59.2% of patients had bilateral hilar and mediastinal lymphadenopathy on CT imaging, a feature more consistent with either malignancy or other granulomatous diseases, such as sarcoidosis. A surprisingly low number of patients recalled a definite contact with a known case of TB. In contrast, Jacob et al. described a positive contact history in 75% in their study in non-cancer patients from endemic and non-endemic regions residing in UK at the time of diagnosis [8]. The microbiological diagnostic yield of simple bronchial washings in such patients was determined to be low; around 9.3%, similar to that reported by Ayed and Behbehani in their study, which was around 10% [20].

Cytopathology and microbiological investigations go hand-in-hand for establishing the diagnosis of MTBLA, however in previous studies, the diagnostic yield of cytopathology is reported to be somewhat higher. Navani et al., in their multi-center study on non-cancer MTBLA patients described the diagnostic yield of cytopathology and microbiological investigations to be around 86% and 53%, respectively [11]. They determined necrotizing granulomatous inflammation in 44%, non-necrotizing granulomatous inflammation in 37% of MTBLA patients, while 12% and 2% of their patients had a reactive and inadequate cytology, respectively [11]. In one such study on patients belonging to a TB endemic demographic, necrotizing granulomatous inflammation was described in 49.6%, non-necrotizing granulomatous in 32% MTBLA patients, and an overall sensitivity of cytopathology to be around 97%. The same study reported a sensitivity of 86% for microbiological diagnosis, but did not report this separately for TB culture and Gene Xpert [21]. In our study, 27 (50%) patients had non-necrotizing granulomatous cytology, 14 (25.9%) patients had necrotizing granulomatous cytology (PPV 100%), while 13 (24%) patients diagnosed with MTBLA had non-granulomatous cytology, including one patient with malignant cytology. Thus, the diagnostic yield was 70.3% for granulomatous cytopathology alone, which was lower than that previously determined for non-cancer patients [11,12,21]. On the other hand, the diagnostic yield was 64.8% for positive microbiology (TB culture and Gene Xpert), higher than [11,21], or nearly equal to [12], that determined previously in non-cancer patients previously. The reason for reduced yield and sensitivity of cytopathology for such patients is undetermined; however, the authors postulate that alteration of lymph node cytology due to underlying malignancy, as well as other effects of chemotherapy and radiotherapy may be the possible reasons behind the lower diagnostic yield.

TB culture has been the mainstay for microbiological diagnosis for MTBLA, with a yield between 47% and 62%, as documented in different studies [11,12]. Previous studies also concur that culture and gene Xpert results together improve the overall diagnostic yield of EBUS for MTBLA, in non-cancer patients [11]. Our study estimates a slightly higher sensitivity for Gene Xpert (53%) compared to TB culture (48%) in this regard, however Gene Xpert was performed in a fraction of the total patients in our study. It is noteworthy that MTB Gene Xpert established the diagnosis of MTBLA in eight patients with negative TB culture, while six patients with negative gene Xpert had a positive TB culture result. A higher sensitivity for MTB Gene Xpert, coupled with significantly better reporting time (six to eight hours compared to at least three weeks for TB culture) makes this investigation even more advantageous, particularly for cancer patients, who may benefit from prompt diagnosis and initiation of treatment. The sensitivity of Gene Xpert has previously been reported to be 56% by Mondoni et al. [22] and 46% by Eom et al. [23] in non-cancer patients. No doubt, the overall diagnostic yield would be higher with concomitant use of both TB culture and Gene Xpert, and there are insufficient grounds to write off the importance of one investigation, over the other.

The incidence of mediastinal TB among cancers of different origins has been less often documented. Previous studies indicate that as a disease, TB is more prevalent in patients with hematological than solid organ malignancy [24,25]. There is dearth of epidemiological data with regards to prevalence of mediastinal TB alone, especially from regions of high endemicity of TB. From this point of view, our study provides some pioneering data. As revealed in the study, the incidence was higher in gastrointestinal malignancies overall. Similarly, a higher incidence was seen in patients with endometrial carcinomas (29%), ovarian carcinomas (27%) and papillary thyroid carcinomas (33%).

In the end, the authors wish to acknowledge certain limitations of this study. This study was retrospective in nature, and hence, the predictive values of the diagnostic parameters may have been over-estimated. While radiographic features of MTBLA on CT chest imaging have been described, the sonographic features of the lymph nodes on EBUS have not been mentioned, reason being lack of documented reporting in all patients, on behalf of the original proceduralist. More importantly, the study relied on clinical treatment response and subsequent one year clinical follow-up to estimate the sensitivity, specificity and predictive values for EBUS as against the standard comparison with the presumed gold standard of surgical lymph node biopsy. This approach was mainly a result of logistic and patient-related concerns, which precluded major surgery for each TB negative patient, of our cancer-predominant study population. The authors however think that the approach is justifiable, on grounds of evidence from a number of previous studies, which convincingly state that EBUS carries accuracy comparable to invasive surgical techniques to establish the diagnosis of mediastinal lymphadenopathy, with considerably less morbidity [26,27]. While keeping patients on close clinical follow-up, our approach may be as close to the ideal standard, and would certainly avoid invasive, and quite often unnecessary investigations, to pursue a diagnosis in TB-negative cancer patients. Regarding this as an important limitation, the authors concur with the need of further prospective studies on MTBLA patients to consolidate confidence in this approach.

## Conclusions

Mediastinal tuberculosis can often be an asymptomatic and occult entity in cancer patients, with diverse radiological features, difficult to distinguish from malignant disease on both CT and PET imaging. Diagnostic testing via EBUS-TBNA is minimally invasive, safe, as well as accurate for establishing diagnosis in these patients. The diagnostic yield and sensitivity of granulomatous nodal cytopathology appears to be lower in cancer compared to non-cancer patients, and concurrence of malignant disease and MTBLA is rare, but possible. The diagnostic yield of microbiological investigations on the other hand is somewhat better, and in this regard, MTB Gene Xpert assay appears to have a slightly higher sensitivity than routine TB culture. Finally, there is significant disease burden of mediastinal tuberculosis in cancer patients of a TB endemic demographic, and relatively higher in those with a primary GI malignancy.
